# Complement Activation and Inhibition in Wound Healing

**DOI:** 10.1155/2012/534291

**Published:** 2012-12-30

**Authors:** Gwendolyn Cazander, Gerrolt N. Jukema, Peter H. Nibbering

**Affiliations:** ^1^Department of Surgery, Leiden University Medical Center, Albinusdreef 2, 2333 ZA Leiden, The Netherlands; ^2^Department of Surgery, Bronovo Hospital, 2597 AX The Hague, The Netherlands; ^3^Department of Trauma Surgery, University Hospital Zurich, Rämistrasse 100, 8006 Zürich, Switzerland; ^4^Department of Infectious Diseases, Leiden University Medical Center, Albinusdreef 2, 2333 ZA Leiden, The Netherlands

## Abstract

Complement activation is needed to restore tissue injury; however, inappropriate activation of complement, as seen in chronic wounds can cause cell death and enhance inflammation, thus contributing to further injury and impaired wound healing. Therefore, attenuation of complement activation by specific inhibitors is considered as an innovative wound care strategy. Currently, the effects of several complement inhibitors, for example, the C3 inhibitor compstatin and several C1 and C5 inhibitors, are under investigation in patients with complement-mediated diseases. Although (pre)clinical research into the effects of these complement inhibitors on wound healing is limited, available data indicate that reduction of complement activation can improve wound healing. Moreover, medicine may take advantage of safe and effective agents that are produced by various microorganisms, symbionts, for example, medicinal maggots, and plants to attenuate complement activation. To conclude, for the development of new wound care strategies, (pre)clinical studies into the roles of complement and the effects of application of complement inhibitors in wound healing are required.

## 1. Introduction

### 1.1. Wound Healing

Wound healing is often completed within two weeks after injury, although tissue remodeling may take several months up to two years. The process of wound healing consists of three, overlapping phases, that is, inflammation, tissue proliferation and tissue remodeling [1–3]. During the different phases, a complex series of sequential cellular and biochemical responses, which are described in some detail in Section 1.2, restores the injured tissue.

Chronic wounds occur in individuals having defects that either prevent the healing process or allow healing to continue without leading to a proper anatomical and functional result. Risk factors for the development of chronic wounds include vascular diseases, diabetes mellitus, pressure (necrosis), alcohol and nicotins abuse, and old age [2]. Current therapies for chronic wounds include debridement, reduction of bacterial load, pressure offloading, topical negative pressure, a variety of wound dressings, skin grafting, and reconstructive tissue flaps [4, 5]. However, the outcome of these therapies is unsatisfactory in up to 50% of chronic (present for one year) wounds [6], resulting in significant morbidity and mortality to patients. Development of new therapies that promote the healing of chronic wounds is therefore an important area of current research. A potential new treatment could be cellular therapy with bone marrow-derived mesenchymal stem cells [6, 7]. Other promising strategies involve the application of anti-inflammatory agents, for example, complement inhibitors, as persistent inflammation is often key to impaired wound healing [2, 8, 9].

### 1.2. Cellular and Molecular Processes Restore Injured Tissues

Tissue injury immediately initiates an array of physiological processes that lead to wound repair and regeneration. Although the exact underlying mechanisms of action are unclear, it is known that the immune systems play an essential role in the regulation of these processes [1–3]. Instantly after tissue injury, damage-associated molecules, such as S100 and the high mobility group box 1 (HBGM1) proteins, defensins, lectins, cardiolipin, cellular DNA and dsRNA, and even intact mitochondria, occur in the extracellular microenvironment. Interaction of these molecules with multiligand receptors, such as toll-like receptors (TLRs) and C-type lectins, on surfaces of tissue and immune cells activate the cellular and molecular effector mechanisms of the innate immune system, including activation of the clotting and complement system, acute phase protein and pentraxin production, and the cellular inflammatory responses [10]. 

Following blood capillary vessel injury, an immediate reflex promotes vasoconstriction, slowdown of blood flow, and the local formation of a platelet clot. In addition, injured tissue cells release factors that stimulate the formation of a fibrin clot (containing a.o. fibronectin and vitronectin), that traps blood cells including platelets and red blood cells. This provisional extracellular matrix allows tissue cells to migrate to the wound area. The activated kallikrein-kinin system provides vasoactive kinins that mediate vasodilation and increased vascular permeability. The complement system is activated by distinct carbohydrate and lipid residues on altered self-molecules and injured cells and the cellular inflammatory response is subsequently initiated. Neutrophils are the first inflammatory cells that migrate into wounds to debride necrotic and apoptotic cells and eliminate infectious agents from the wound bed [3]. Gradually neutrophils are replaced by monocytes that exert the same scavenging activities. Monocytes at the wound site will also develop into macrophages that produce an array of inflammatory molecules, including chemokines, anti-inflammatory mediators, enzymes (proteolytic enzymes, metalloproteases), reactive oxygen species, and growth factors. A major drawback of infiltration of activated phagocytes is their ability to produce and release reactive oxygen species and proteolytic proteases that exert detrimental effects on healthy tissue cells [3]. In addition, immature dendritic cells collect antigens, for example, altered self-antigens, at the site of the wound and transport them to the draining lymph nodes where the dendritic cells mature and instruct T cells become effector cells. 

The chemotactic mediators and growth factors produced by macrophages and healthy bystander cells stimulate angiogenesis and attract endothelial cells and fibroblasts that contribute to the proliferative phase of wound healing [3]. Simultaneously, effector T lymphocytes migrate to the wound and play a regulatory role in wound healing and collagen levels [3]. During the remodeling phase of the healing process, redundant cells die by apoptosis and collagen is remodeled and realigned. While the functions of the cells involved in the healing processes have been reported in much more detail than that described above, the biochemical responses leading to the activation of these cells at the site of injury are not widely investigated. However, it is well known that activation of the complement system is crucial in regulating the cellular responses in innate immunity. 

### 1.3. Aims of This Paper

As described above, the first response to tissue injury is characterized by activation of the cellular and molecular effectors of the innate immune system, including the complement system. However, inappropriate complement activation, for example, in chronic wounds, will result in detrimental effects due to its ability to induce cell death and promote prolonged inflammation [10, 11]. Experiments in animals with deficiencies in complement components indicate that attenuation of complement activation promotes wound healing [12–19]. Therefore, complement inhibitors are considered as candidates for development of novel therapeutic agents for chronic nonhealing wounds. 

Based on these considerations, this paper focuses on (1) the current understanding of the dual roles of complement activation in wound healing and (2) the present and novel complement inhibitors to be considered for treatment of chronic wounds. 

## 2. Overview of the Complement Pathways and Their Functions in Wounds

### 2.1. The Complement System

**Figure 1:**
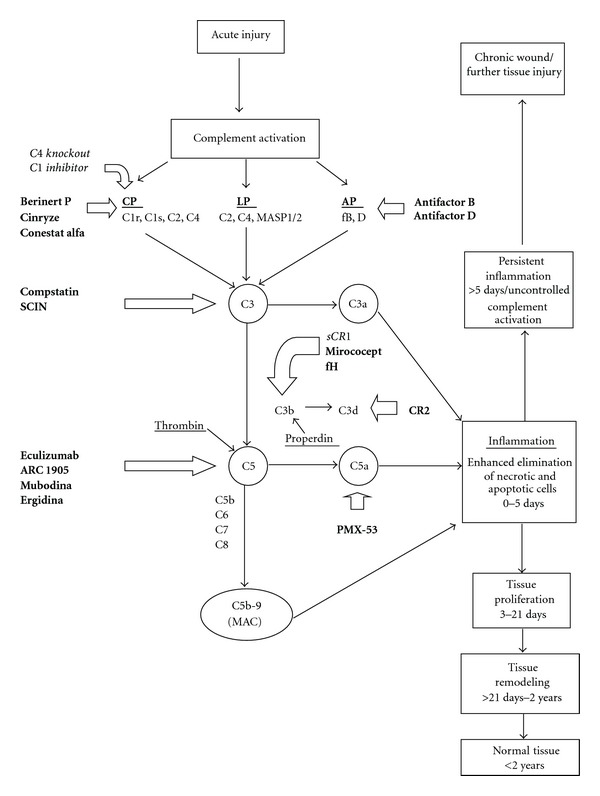
A simplified overview of the complement activation cascade after injury leading to wound healing. Three major pathways of complement activation, that is, the classical pathway (CP), the alternative pathway (LP), and the lectin pathway (LP), and two minor pathways initiated by properdin and thrombin are known. C is a complement component, MASP is mannan-binding serine peptidase, fB and D are factors B and D, SCIN is staphylococcal complement inhibitor, sCR1 is soluble complement receptor 1, fH is factor H, CR2 is complement receptor 2 and MAC is membrane attack complex. For simplicity, not all of the natural regulators of complement activation are shown in this diagram. The (pre)clinical complement inhibitors are denoted in bold and the complement factors that have been investigated in burn wound models in italic. C1 inhibitor affects C1r, C1s from the CP, and MASP 1 and MASP 2 from the LP. C4 knockout also affects both CP and LP.

The activated complement system is a crucial effector mechanism of the innate immune response to tissue injury. In general, the complement system can be activated by a number of pathways: the classical pathway (by immune complexes), the lectin pathway (by mannose residues and ficolins), and the alternative pathway (by spontaneous activation and microbial structures) and by properdin and thrombin [20]. The result of activation of any of these pathways is cleavage of the central factor C3 into C3a and C3b by C3 convertase (except thrombin, which activates the cleavage of C5 by C5 convertase) [21]. Thereafter, the terminal pathway of the complement system with factors C5b to C9 is completed (Figure 1). These latter factors form the membrane attack complex (MAC), which creates pores in the microbial cell wall resulting in cell lysis. C3a and C5a are the most important chemoattractants that are produced as part of the activation of the complement system. In addition, recognition of necrotic and apoptotic cells by activated complement components leads to the deposition of complement components, such as C3-fragments, on their membrane, which promotes phagocytosis and elimination of the damaged cells by phagocytic cells and also results in the generation of the MAC on these damaged cells. The major drawback of complement activation is that the tolerance against self-molecules can be broken, leading to responses to these self-molecules and, as a consequence, to further tissue injury and impairment of wound healing (Figure 1). Fortunately, host cells are protected from complement-mediated injury by fluid phase and membrane-bound regulators of complement activation, such as factor B, factor D, factor I, CD35, CD46, CD55, and CD59 [22, 23]. However, during tissue injury, the expression of these complement regulators may be decreased, resulting in reduced protection of the cells and increased tissue damage. Together, while complement activation is needed to restore tissue injury, inappropriate complement activation can cause injury and contribute to further tissue damage [11]. 

### 2.2. Roles of Complement in Wound Healing

There are a few studies that report beneficial effects of complement-activating components on wound healing. First, Strey et al. reported that complement C3a and C5a are absolutely required for liver repair in a mouse model of liver injury [24]. Second, Bossi et al. topically applied C1q, vascular endothelial growth factor, or saline on wounds in rats and after 2 weeks vessel formation was examined [25]. Results revealed that animals treated with C1q and vascular endothelial growth factor exhibited increased numbers of new vessels as compared to control animals. In addition, application of C1q resulted in increased permeability, proliferation, and chemotaxis of endothelial cells, indicating that C1q has proangiogenic activity and thus can promote wound healing [25]. Third, topical application of C3 (100 nM) on a rat wound model resulted in a 74% increase in maximum wound strength as compared to control rats [26]. Also, inflammatory cells, fibroblast migration and collagen deposition in the wounds were enhanced in the C3-treated mice as compared to control animals. Despite the positive effects of C1q or C3 application on wound healing in these models of acute injury, the possibility that complement components exert an entirely different, that is, detrimental, effect on chronic wounds is likely. In agreement, in the majority of chronic wounds, MAC deposition is found at the ulcer margin, but not in the intact skin [27]. It has also been shown that patients with chronic leg ulcers have increased serum levels of C3 [28, 29]. 

While enhanced levels of complement activating factors are found in chronic wounds, it is interesting to study the outcomes of wounds in which complement activation is attenuated. It has been shown that animals with a genetic complement deficiency or individuals treated with a complement inhibitor are protected from the symptoms resulting from chronic inflammatory processes [12–17]. Interestingly, Wahl et al. published a study regarding the effect of complement depletion by cobra venom factor (CVF) on healing of acute wounds in guinea pigs [13]. CVF forms a stable complex with Bb resulting in continuously activated C3/C5 convertase [14], resulting in depletion of complement activity, while it is resistant to complement regulatory factors, such as factor H and I. CVF was administered intraperitoneally to guinea pigs over a 24-hour period while control animals received the diluent of CVF. After 24 hours, the wound exudates from the complement-depleted pigs showed a 50% reduction in infiltrating neutrophils and four times more erythrocytes than exudates from control animals. Wound debridement, fibroblast proliferation, connective tissue formation, and capillary regeneration did not differ between CVF-treated and control, wounded animals. It should be realized that only acute wound healing was investigated and that CVF could have had other systemic effects that affected wound healing in the guinea pigs. In this connection, it has been described that additional injections of CVF were administered and that these guinea pigs developed lethargy, leucopenia, and loss of weight. Unfortunately, no definitive conclusion as to the role of complement in wound healing can be drawn from these data. Furthermore, CVF initially is a complement activator, which can induce tissue damage instead of repair. Together, complement components play opposite roles in acute and chronic wounds.

### 2.3. Roles of Complement in Burn Wounds

Studies by Van de Goot et al. into the roles of complement in burn wounds showed enhanced levels of complement degradation factor C3d, indicative of complement activation, in the wound [30]. C3d remains elevated in the wound until 46 days after the burn injury. The amount of the acute phase reactant C-reactive protein and the influx of neutrophils and macrophages were also higher in the wounds during this period and indicate the persisting inflammation. Machens et al. compared the amount of C3a in wound fluids from a group of patients younger than 60 years and from a group older than 60 years with deep second-degree burn wounds [31]. Results revealed elevated C3a levels in both groups during the first 24 hours after thermal injury. However, thereafter the C3a levels in the wound fluid decreased in the young group, but not in the group with the older patients, indicating that persistent complement activation is associated with the delayed wound healing in the older patients. In agreement, others reported elevated serum levels of C3 and C3d in patients with burn wounds and these levels correlated with the severity of the trauma and the clinical outcome [32]. Furthermore, Mulligan et al. found that intravenous injection of soluble human recombinant complement receptor type 1 (sCR1) at 5 and 15 minutes and at 1 and 4 hours after thermal injury into rats resulted in decreased dermal vascular permeability and water content and reduced recruitment and activation of neutrophils in wound biopsies as compared to the biopsies from control rats [15]. The sCR1-treated rats were protected against complement-dependent tissue injury. In another study, the effects of a C1 inhibitor intravenously administrated immediately after thermal injury on progression of the depth of fresh burn wounds in pigs were assessed [16]. In contrast to the control group, the lower dermal vascular network was not altered in the C1 inhibitor treatment group and there was only activation of endothelial cells in the subepidermal and mid-dermal layer. Whereas in the control group there was necrosis of the lower dermal zones, these zones were normal in the C1 inhibitor group. As most studies focused on the short-term effects of complement inhibitors on wound healing, Begieneman et al. determined the effects of 14 daily intravenous administrations of C1 esterase inhibitor on wound progression in dorsal full-thickness burn wounds in rats [17]. Results revealed that the C1 inhibitor reduced the amount of granulation tissue and macrophage infiltration in these animals. The amounts of complement factors C3 and C4 in the wounds were lower (although not significant) in the C1 inhibitor-treated group than in the control group. Furthermore, the C1 inhibitor did enhance reepithelialization. The data from this study show that systemic administration with C1 inhibitor improves healing in burn wounds. In addition, Radke et al. demonstrated in a pig burn wound model that inhibition of C1 is beneficial for the clinical outcome, as indicated by vital signs and reduced edema formation, and C1 inhibitor diminished bacterial translocation [33]. Finally, Suber et al. found reduced burn wound depth and neutrophil migration in C4 knockout mice as compared to wild type animals [18]. Burn wounds in C4-deficient mice healed without contracture, scar formation, or hair loss in contrast to the wild type mice. Moreover, the severity of the burn wound was significantly less in C4 knockout mice than in wild type animals. Together, both in preclinical and animal studies, attenuation of complement activation stimulates the wound healing process. Therefore, the various potential complement-inhibiting agents and their therapeutic effects are discussed in the next section.

## 3. Exogenous Complement Inhibitors

### 3.1. Current (Pre)Clinical Complement Inhibitors

In clinical practice, only a few complement inhibitors are currently available (Table 1). Plasma-derived human C1 inhibitors berinert P and cinryze and the recombinant human C1 inhibitor conestat alfa are currently applied in patients suffering from hereditary angioedema (HAE) [34, 35]. Furthermore, C5 inhibitor eculizumab is used in patients with paroxysmal nocturnal hematuria (PNH) [36]. An overview of these and other (pre)clinical complement inhibitors and their interaction with the complement system is given in Table 1 and Figure 1.

Recently, the C5 inhibitor pexelizumab failed in a Phase III study as it did not reduce infarction and mortality in patients after coronary intervention [37]. Pexelizumab inhibited both C5a and MAC formation *in vitro*, while *in vivo* only C5a was reduced with minimal effects on inflammation and risk biomarkers. Compstatin (POT-4), isolated from a phage-displayed random peptide library, is the only C3 inhibitor under investigation in Phase II studies for the treatment of acute macular degeneration (AMD) [38]. Compstatin is also tested in preclinical experiments for possible applications in PNH, sepsis, transplantation, and cancer. Furthermore, Mirococept (APT070), a membrane-targeted myristoylated peptidyl construct derived from soluble complement receptor 1, is currently examined in a multicenter, double-blind, randomized, case-control study for prevention of ischemia-reperfusion injury in cadaveric kidneys for transplantations [39, 40]. Anticomplement factor D is analyzed in a Phase II study in patients with AMD [36]. However, the Phase II study with C5a-inhibitor PMX-53 in AMD patients was discontinued because of lack of success. Nevertheless, this inhibitor is still under investigation for the use in osteoarthritis. 

Phase I studies are performed with targeted factor H (TT30), that is, factor H coupled to CR2, for AMD and PNH [41]. This targeted inhibitor binds to C3b/C3d coated cells and blocks assembly of C3 and C5 convertases. Various other complement inhibitors coupled to CR2 were tested in patients with chronic glomerulonephritis [42]. In addition, the C5 inhibitor eculizumab, which is already approved by the FDA for PNH, was also tested as treatment for several other diseases, including kidney transplants and haemolytic uraemic syndrome (HUS) [36]. The anti-C5 aptamer ARC 1905 is investigated for its potential use in AMD [36]. Finally, the effects of plasma-derived factor H concentrate, anti-complement factor B (TA106) and C5 inhibitors, such as mubodina and ergidina, in complement-mediated diseases were evaluated in preclinical studies [36].

### 3.2. Medicinal Maggots Produce Complement Inhibitors

Larvae of medicinal maggots (*Lucilia sericata)* are successfully used to heal severe, infected acute and chronic wounds in the clinical practice [43–46], and in 2004, Maggot Debridement Therapy (MDT) was approved by the US Food and Drug Administration (510[k] no. 33391) [47]. Our current research focuses on the mechanisms underlying the beneficial actions of maggots on wound healing. So far, maggot excretions/secretions (ES) in therapeutic concentration ranges lack direct antibacterial properties [48] but inhibit biofilm formation and multiple proinflammatory responses [49, 50], which could explain part of the mechanism of action of maggots in wound healing. Others reported beneficial effects of maggot ES on the modulation of extracellular matrix components leading to enhanced tissue formation and accelerated healing [51, 52]. 

**Figure 2:**
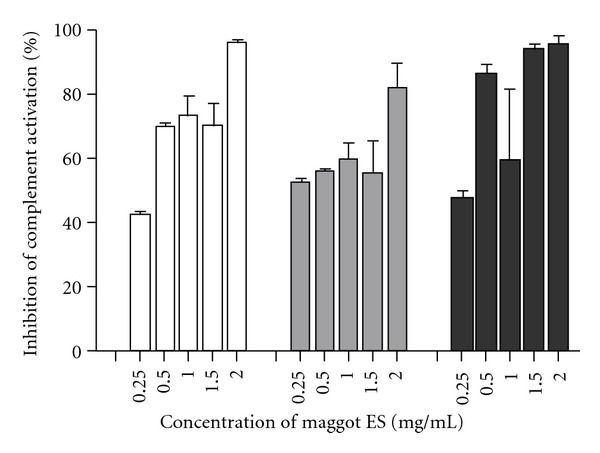
Dose-dependent effect of fresh collected maggot ES on activation of the classical pathway (white bars), the alternative pathway (grey bars), and the lectin pathway (black bars) in normal human sera. The complement activation in four different sera was determined with the enzyme immunoassays from Wieslab (EuroDiagnostica BV, Arnhem, The Netherlands) according to manufacturer's instructions. The percentages inhibition was calculated using the values in the sera without maggot ES as 0%. The results are means and SD of four independent experiments.

 Recently, we found that maggot ES efficiently reduced complement activation in normal and immune-activated sera in a dose-dependent fashion with maximal inhibition of 99.9% (Figure 2) [53]. Most likely, ES degrade individual complement components, at least C3 and C4, in a cation-independent manner. Consumption of complement components via ES-mediated initiation of the complement cascade has been ruled out. The complement inhibitory molecule(s) in maggot ES proved to be temperature- and protease-resistant. Together, attenuation of complement activation by ES may contribute to the improved wound healing that is observed during MDT in the clinical practice [43–46]. As maggots and their ES are well tolerated by patients, it can be envisaged that the complement inhibitory molecules within ES are potential candidates for the development of novel complement inhibitors.

### 3.3. Complement Inhibitors Produced by Other Symbionts

As the complement system is a rapid and effective defense system, practically each successful microorganism has developed strategies and molecules to evade the actions of complement [54, 55]. Therefore, it is virtually impossible to give a brief, complete overview of all complement inhibitors produced by infectious agents described in the literature, but we will show some examples. *Staphylococcus aureus* is one of the pathogens that produces at least seven molecules with complement inhibitory molecules, including C3 inhibiting molecule staphylococcal complement inhibitor (SCIN), which prevents the conversion of C3 by convertases (C3b/Bb and C4b2a) and staphylococcal superantigen-like protein 7 that prevents C5 cleavage [54, 56]. Another example pertains to the herring worm *Anisakis simplex* [57]. Consumption of raw herring can cause intestinal infections by this herring worm, which possesses complement-inhibiting properties to evade the human immune defense. *Anisakis simplex* also excretes biochemical substances that harm the intestines. Therefore, the human immune system evolutionary developed (undefined) strategies against this parasitic infection resulting in death of the herring worm in all immunocompetent patients. Borreliaespecies, causing borreliosis (Lyme disease), also produce complement inhibitors to evade the innate immune system [58, 59]. Binding of a borrelial surface protein to complement factor H limits AP activation and binding to complement inhibitor C4b-binding protein avoids CP activation. However, *Borreliae* appear to have specific effects on the complement cascade which finally do not result in a decrease of the inflammatory response. Adversely, aggravated inflammation is observed during borrelial infection. The scabies mite *Sarcoptes scabiei*, which can cause a parasitic infestation of the skin, expresses serine protease inhibitors in their gut and faeces that interfere with all three complement activation pathways leading to an overall complement inhibition [60]. Probably, the scabies protect themselves by excreting complement inhibitors.

### 3.4. Complement Inhibitors in Medicinal Plant Extracts

Although plants lack genes encoding complement molecules, complement inhibitors have been found in extracts from various species of plants and trees (Table 2). Here, we will only mention some interesting examples from plants used in traditional medicine all over the world to treat (inflammatory) diseases and wounds. Deharo et al. studied complement inhibiting properties of plant extracts used by the Tacana ethnic group in Bolivia and found six new species that produced molecules that inhibited the classical and alternative pathway [71]. Fernández et al. showed complement reducing effects in extracts of five different plants that are traditionally used in Argentina [61]. Hawaiian medicinal plants were investigated by Locher et al. and *Eugenia malaccensis* was found to produce molecules that inhibit the classical pathway, which could explain (in part) its activity against inflammatory diseases, including wound healing [80]. Other examples of plants producing complement inhibitors in Mali are the extracts of the root of *Entada africana*, leaves of *Trichilia emetica* and *Opilia celtidifolia,* and water extract of the aerial parts of *Biophytum petersianum Klotsch,* which are traditionally used in Mali to cure wounds and to reduce fever [65, 73, 79, 90]. Natural latex from rubber trees also has wound healing properties and extracts of *Jatropha multifida* and *Croton Draco* were able to inhibit the classical pathway of complement activation [78, 85]. Plant extracts interfere with the complement system at different stages of the cascade (Table 2). *Bridelia ferruginea*, *Isopyrum thalictroides,* and *Ascophyllum nodosum* inhibit C1 formation and the latter one also forms a complex with C4 [69, 70, 76, 81]. *Glycyrrhiza glabra* reduces C2 [83, 84] and *Glycine max* inhibits synthesis of C2 and C4 [81, 82]. C3 is affected by *Aloe vera*, *Boswellia serrata, Glycyrrhiza glabra, Rosmarinus officinalis,* and *Ulex europaeus* [62, 63, 74, 75, 81, 83, 84, 88, 89, 91]. Production of anaphylatoxin C5a is decreased by *Piper kadsura* and *Rosmarinus officinalis* [81, 86, 88, 89]. Future research should focus on the purification and characterization of the effective substances in plants and the specificity and exact mechanisms of action of these compounds. 

## 4. Discussion and Future Research

Complement serves as a rapid and efficient immune surveillance system to control infection and tissue injury. The complement system regulates the clearance of necrotic and apoptotic cells, inflammation, and tissue regeneration. However, elevated levels of C3, C3a, C3d, and MAC have been reported in chronic wounds and burn or traumatic wounds [27–32], indicating that uncontrolled complement activation occurs in such wounds. In addition, studies in animals with deficiencies in complement components and in patients treated with complement inhibiting agents confirmed the importance of controlling the complement system in wound healing and fibrosis [12–18, 108]. Specific inhibitors can balance the functional activities of the complement system and progress the healing process, as shown in patients with burn wounds treated with a C1 inhibitor or a soluble human recombinant complement receptor type 1 as well as in C4-deficient mice [15–18]. Thus, attenuation of complement activation by therapeutic agents may improve the healing process in chronic wounds.

However, several challenges have to be overcome before complement inhibitors can be included in the therapeutic arsenal for wound care. For example, complement inhibitors should act locally at the site of inflammation or injury, thus avoiding the adverse effects of a systemic complement blockade, that is, infection and impaired wound healing [109]. For this purpose, current research focuses on the development of strategies to target the complement inhibitor to the sites of complement activation, regardless of the location. In this connection, a Phase I study has recently been performed in which various complement inhibitors were linked to a targeting moiety consisting of complement receptor 2 (CR2) [110]. CR2 binds long-lived C3 fragments and thereby acts to target the attached complement inhibitor to the site of inflammation/injury. In agreement, experiments in mice showed an increased potency and prolonged local presence of such complement inhibitors, while leaving the systemic complement activation intact [42]. No increased risk of infection or sepsis was observed in these animals. Another example is perfusion of cadaveric kidneys during the transfer from the donor to the recipient with mirococept which is a peptidyl derivative of sCR1 engineered to stick to the organ during this process [40]. 

One more issue pertains to the contribution of local production and functional activities of complement components and their regulators. Although the liver is the main source of complement components, the production of several complement components, for example, properdin, C1, C3, and C7, at sites of inflammation/injury should be studied in more detail. Furthermore, good affinity of the complement inhibitors for the target and selectivity are important factors to consider in anti-complement therapies. Moreover, the complement inhibitor must have a long half-life. 

The choice of the complement inhibitor depends on the role the complement has in the disease. C5 inhibitors are preferred for the treatment of diseases in which C5a and MAC play a major role, for example, in HUS and in patients suffering from an infection with the EHEC bacterium [111]. Cleavage of C5 generates C5a, a major inflammatory mediator, and C5b initiating the formation of MAC. These two factors are the key effectors of the complement system responsible for both wound repair and persistent inflammation [112]. Obviously, the effects of complement inhibitors also depend on the stage of the disease in the patients. In this context, it is interesting to see that the C5-inhibitor eculizumab is efficacious for PNH and HUS [36], while pexelizumab having the same mode of action as eculizumab was ineffective in patients undergoing percutaneous coronary intervention after myocardial infarction [37]. This failure of pexelizumab could be due to late administration of the antibody after ischemia-reperfusion and/or differences in their half-lifes, that is, eculizumab has an average half-life of 272 hours and pexelizumab of 7–14 hours. In agreement, administration of pexelizumab before coronary arterial bypass grafting did have a beneficial outcome [37]. Of note, it was found that *in vitro* both C5a and MAC were both blocked by these antibodies directed against C5 while *in vivo* C5a activity (but not MAC) was blocked. Finally, there are concerns about the clinical use of nonspecific complement inhibiting agents as these agents may have adverse consequences for patients, such as (recurrent) infections [109]. 

Although there are a lot of challenges to overcome, there are some promising complement inhibitors. For example, the pathway-independent inhibitor compstatin is extensively tested in clinical studies in patients suffering from acute and chronic inflammatory conditions. The results up to date are successful [36]. Furthermore, a novel complement inhibitor could be based on the active component(s) in ES of *Lucilia sericata* larvae as ES reduce all three complement activating pathways in normal and immune-activated human sera in a dose-dependent manner [53]. Moreover, it should be kept in mind that these maggots are already in clinical use for many years without any side effects reported in the literature nor in our own clinical experience with this therapy over the past ten years [44, 45]. 

Another important question that remains unanswered is how much the complement system can be attenuated without the risk of loss of protection. Based on our finding that a single maggot produces approximately 2 *μ*g of ES per hour [53, 113] and assuming that 125 maggots are applied on a wound surface (of about 25 square centimeter), the amount of ES in the wound (per hour) is 250 *μ*g, which correlates with a 50% complement reduction (Figure 2). Thus, we believe that reduction of the local complement activity of about 50% is safe and effective. However, further research is required before a definitive conclusion can be drawn.

To conclude, well-designed (pre)clinical studies aimed at understanding the roles of complement in the pathology of chronic wounds, with the hope of innovative drugs and their clinical implementation to promote healing in patients with chronic wounds, are urgently needed.

## Figures and Tables

**Table 1:** An overview of (pre)clinical complement inhibitors.

Complement inhibitor	Medicine	Diseases	Study phase
Recombinant C1 inhibitor	Conestat alfa	HAE Side effects: headache and allergy.	In clinical use, EU approved.
	(Ruconest in Europe/Rhucin in USA)		

Plasma-derived C1 inhibitors	Berinert P/cinryze	HAE	In clinical use, FDA approved.

C3 inhibitors	Compstatin (POT-4)	AMD	Phase II
	Staphylococcal complement inhibitor (SCIN)		Preclinical

Myristoylated peptidyl derived from soluble CR1	Mirococept (APT070)	Delayed graft function of cadaveric kidney after transplantation.	Phase II

Factor H	Plasma-derived factor H concentrate	HUS, AMD	Preclinical
	TT30/targeted alternative pathway inhibitor/factor H	PNH, AMD	Phase I

Factor D inhibitor	Anticomplement factor D	AMD	Phase II

Factor B inhibitor	TA106/anti-complement factor B	AMD	Preclinical

C5 inhibitors	Eculizumab	PNH Side effects: headache, thrombocytopenia, gastrointestinal complaints and infections. Before use: vaccination against meningococcal infection.	In clinical use, FDA approved.
		Various other diseases, for example, kidney transplants, HUS, AMD.	Phase I
	Pexelizumab		Phase III study failed
	Mubodina	HUS	Preclinical
	Ergidina	Ischemia/reperfusion injury	Preclinical
	ARC 1905	AMD	Phase I

C5a inhibitor	PMX 53 and several other compounds	AMD	Phase II study discontinued
		Osteoarthritis	Phase I

Targeted complement inhibitors	Targeted (CR2 mediated) complement inhibitors	Chronic glomerulonephritis	Phase I

HAE: hereditary angioedema; AMD: acute macular degeneration; HUS: haemolytic uraemic syndrome; PNH: paroxysmal nocturnal haematuria.

**Table 2:** An overview of complement inhibitors in extracts from plant species.

Plant L.	Part of plant (extract)	Mode of action	Beneficial effects	References
*Achyrocline flaccida* (Yellow Marcela)	Aerial parts	CP inhibition. IC_50_ (CP) = 23.5–88.9 *μ*g/mL	Antispasmodic, antipyretic, antihelmintic, antibacterial, antiviral. Stimulant, emmenagogue, excitant.	[61]

*Aloe vera *	Leaves	AP activation, resulting in consumption of C3.	Antibacterial, antifungal, antiparasitic, antitumor, laxative. Used for seborrheic dermatitis, radiation dermatitis, psoriasis vulgaris, genital herpes, burn wounds, diabetes, HIV infection, ulcerative colitis, pressure ulcers, mucositis, aphthous stomatitis, acne vulgaris, lichen planus, frostbite, alopecia, systemic lupus erythematosus, arthritis, tic douloureux.	[62, 63]

*Apeiba tibourbou* (Monkey comb)	Leaves	CP and AP inhibition.	Antispasmodic, mucilaginous, and pectoral properties. Used for rheumatism.	[64]

*Artemisia species* *(A. dracunculus, A. montana, A. princeps, A. rubripes, A. tripartita) *	Leaves	CP inhibition. IC_50_ (CP) = 54.3–64.2 *μ*g/mL	Used for colic pain, vomiting, diarrhea, dysmenorrhea.	[65–68]

*Ascophyllum nodosum* (Brown seaweed)	Leaves	CP inhibition. Fucoidan binds C1q and prevents the formation of active C1. It forms a complex with C4	Anti-inflammatory, antiangiogenic, anticoagulant, antiadhesive.	[69, 70]

*Astronium urundeuva *	Stem bark	CP and AP inhibition. IC_50 _ (CP) = 64 *μ*g/mL IC_50 _ (AP) = 111 *μ*g/mL	Used for wound healing, bone healing, inflamed sores, gastric ulcers, uterine hemorrhages, metrorragias, cervicitis.	[71]

*Avicennia marina* (Evergreen shrub)	Stem bark	CP inhibition. IC_50 _ (CP) = 23–248 *μ*g/mL	Antitumor, anti-inflammatory, antiviral. Used for skin diseases, wound healing, rheumatism, smallpox, ulcers, malaria.	[72]

*Biophytum petersianum Klotsch *	Aerial parts	CP inhibition. IC_50 _ (CP) ≤2–86 *μ*g/mL	Used for wound healing, inflammation.	[65, 73]

*Boswellia serrata* (Frankincense)	Oleogum resin	CP inhibition, it inhibits C3 convertase	Antihelminthic, antiseptic, haemostatic, analgesic, cardiotonic, diuretic, aphrodisiac, laxative. Used for Crohn's disease, ulcerative colitis, bronchial asthma, rheumatoid arthritis, osteoarthritis, wound cleaning, reducing fat, diarrhea, improving menstruation.	[74, 75]

*Bridelia ferruginea *	Stem bark	CP and AP inhibition. Inhibition of C1 and terminal complex.	Used for rheumatism.	[76]

*Cochlospermum vitifolium *(silk cotton tree)	Stem bark	CP and AP inhibition. IC_50 _ (CP) = 104 *μ*g/mL IC_50 _ (AP) = 135 *μ*g/mL	Used for diabetes, hepatobiliary and cardiovascular diseases, hypertension, pain, kidney diseases, ulcers.	[71, 77]

*Croton draco *	Latex	CP and AP inhibition. IC_50 _ (CP) = 430–590 *μ*g/mL IC_50 _ (AP) = 680–930 *μ*g/mL	Antibacterial, antitumor, antiviral. Used for wound healing, inflammation.	[78]

*Entada africana *	Roots	CP inhibition. IC_50 _ (CP) = 75–370 *μ*g/mL	Hepatoprotective, haemostatic, antipyretic, antiseptic, diuretic, antigonococci, antisyphilitic, antiparasitic, abortifacient. Used for wound healing, malaria, respiratory diseases, psoriasis, rheumatism, cataract, dysentery.	[79]

*Eugenia malaccensis* (Malay rose apple)	Stem bark	CP inhibition: IC_50 _ (CP) = 12 *μ*g/mL AP was activated: 50 % activation at 6 *μ*g/mL	Used for general debility, sore throat, wound healing, candidiasis, venereal diseases, tuberculosis, digestive tract disorders.	[80]

*Eupatorium arnottianum *	Aerial parts	CP and AP inhibition. IC_50 _ (CP) = 5.0–155.9 *μ*g/mL IC_50 _ (AP) = 101.3 *μ*g/mL	Antimicrobial, antiviral, antinociceptive. Used for gastric pain.	[61]

*Eupatorium buniifolium *	Aerial parts	CP inhibition. IC_50 _ (CP) = 44.1–66.7 *μ*g/mL	Hepatoprotective, antiviral, antiseptic.	[61]

*Euterpe precatoria* (Açai)	Roots	CP and AP inhibition. IC_50 _ (CP) = 105 *μ*g/mL IC_50 _ (AP) = 147 *μ*g/mL	Antioxidant. Used for muscular pain, sciatic pain, liver and kidney diseases, wound healing, skin ulcers, edema, inflammatory diseases.	[71]

*Glycine max* (Soyabean)	Seeds	*In vitro* it inhibits synthesis and secretion of C2 and C4 by guinea pig peritoneal macrophages	Antioxidant, anti-inflammatory, antitumor, antioestrogenic, antifungal, insulinotropic. Used for atherosclerosis, skin whitening,	[81, 82]

*Glycyrrhiza glabra *(Licorice)	Roots and rhizomes	Glycyrrhizin binds to C3a and C3. It induces conformational changes in C3 and it inhibits CP at the level of C2.	Anti-inflammatory, antiviral, antimicrobial, antioxidative, antitumor, immunomodulatory, hepatoprotective, cardioprotective, diuretic, anabolic, laxative, contraceptive. Used for wound healing, cystitis, diabetes, cough, stomachache, tuberculosis, nefrolitiasis, lung ailment, Addison's disease, gastric ulcers, improvement of voice, improvement of male sexual function.	[83, 84]

*Isopyrum thalictroides *	Roots and aerial parts	CP inhibition. Ca^2+^ and Mg^2+^ dependent complement inhibition. It inhibits C1 formation.	Rheumatism, neuralgia, silicosis, malaria.	[81]

*Jatropha multifida/Jatropha curcas *(Coral plant)	Latex	CP inhibition, mediated by Ca^2+^ depletion	Used for infected wounds.	[81, 85]

*Lithraea molleoides *	Leaves	CP inhibition. IC_50 _ (CP) = 59.0–86.1 *μ*g/mL	Anti-arthritic, haemostatic, diuretic, tonic. Used for respiratory diseases. It causes allergic contact dermatitis.	[61]

*Opilia celtidifolia *	Leaves	CP inhibition. IC_50 _ (CP) = 0.5–29 *μ*g/mL	Haemostatic. Used for wound healing.	[73]

*Piper kadsura* (Japanese pepper)	Stem bark	It inhibits C5a-induced chemotaxis and decreased the stimulated production of TNF-*α* and IL-1-*β*	Asthma, rheumatic arthritis	[86]

*Phyllanthus sellowianus *	Leaves and stems	CP and AP inhibition. IC_50 _ (CP) = 11.2–22.0 *μ*g/mL IC_50 _ (AP) = 280.6 *μ*g/mL	Hypoglycemic, diuretic, laxative, antiseptic, antinociceptic.	[61, 87]

*Rosmarinus officinalis/Melissa officinalis *(Rosemary)	Leaves	CP and AP inhibition. It binds C3 and inhibits C5 convertase. C5a generation is decreased. IC_50 _ (CP) = 2 *μ*g/mL	Antispasmodic, choleretic, hepatoprotective, anti-inflammatory, antitumor, antioxidant. Used for renal colic pain, dysmenorrhea, respiratory disorder (bronchial asthma), stimulation of hair growth, relaxation of smooth muscles of trachea and intestine, peptic ulcers, atherosclerosis, ischaemic heart disease, cataract, improvement of sperm motility.	[81, 88, 89]

*Trichilia emetica* (Natal mahogany)	Leaves	CP inhibition. IC_50 _ (CP) ≤15–62.5 *μ*g/mL	Antipyretic, antiepileptic, antigonococci, antisyphilitic, anti-parasitic. Used for wound healing, dysmenorrhea, asthma, vomiting, hepatitis, improvement of fertility (women), gastric diseases, malaria, hypertension, rheumatism, lumbago.	[90]

*Triplaris americana* (Ant tree)	Stem bark	CP and AP inhibition. IC_50 _ (CP) = 74 *μ*g/mL IC_50 _ (AP) = 89 *μ*g/mL	Antioxidant, parturifacient. Used for metrorragias, diarrhea, stomachache, intestinal worms, leishmaniasis, skin ulcers.	[71]

*Ulex europaeus *(Common gorse)	Seeds	It attenuates MBL binding on human endothelial cells and inhibited C3 deposition. The dcreased LP activation resulted in less complement-dependent neutrophil chemotaxis. IC_50 _ = 10 pmol/L	None.	[91]

*Uncaria tomentosa *(Cat's claw)	Stem bark	CP and AP inhibition. IC_50 _ (CP) = 124 *μ*g/mL IC_50 _ (AP) = 151 *μ*g/mL	Anti-inflammatory, antiviral, immunostimulating, antimutagenic, antioxidant. Used for gastritis, dermic and urogenital inflammations, asthma, rheumatism, irregular menstruation, digestive, liver and kidney diseases, adjuvant therapy for breast cancer.	[71, 92]

CP: classical pathway; AP: alternative pathway; LP: Lectin Pathway; IC_50_: concentration required for 50% complement inhibition. Most of these complement inhibition tests were performed using complement haemolytic activity assays. Compounds in these plant species inhibiting the complement system are; for example; flavonoids, glucosides, polysaccharides, terpenes, iridoids, polymers, peptides, alkaloids, and oils [81]. Other complement inhibitors from plants are found in *Acanthus ilicifolius*[72], *Atractylodes lancea *[73], *Angelica acutiloba* [73, 81, 93], *Azadirachta indica* [81], *Bupleurum falcatum* [94], *Cedrela lilloi* [81], *Centaurium spicatum* [81], *Cochlospermum tinctorium *[95], *Crataegus sinaica* [81], *Crataeva nurvala* [81], *Curcuma longa* [96], *Dendropanax morbifera Leveille* [97], *Glinus oppositifolius* [79], *Juglans mandshurica* [98], *Ligularia taquetii* [99], *Litsea japonica* [100], *Ligustrum vulgare* [81], *Lithospermum euchromum* [81], *Magnolia fargesii* [101], *Melothria maderaspatana* [102], *Morinda morindoides* [81], *Olea europaea* [81], *Osbeckia octandra* [102], *Ocimum basilicum* [66], *Osbeckia aspera* [81], *Panax ginseng* [103], *Paulownia tomentosa var. tomentosa* [104], *Persicaria lapathifolia* [81], *Petasites hybridus *[81], *Phillyrea latifolia* [81], *Phyllanthus debilis* [102], *Picria fel-terrae* [105], *Plantago major* [81], *Sorghum bicolor* [106], *Terminalia amazonia* [71], *Thymus vulgaris* [66], *Tinospora cordifolia* [81], *Trichilia elegans* [90], *Trichilia glabra* [81, 90], *Vernonia Kotschyana* [72, 73, 95], *Wedelia chinensis* [107], and *Woodfoidra fruticosa* [81].
